# Differentiation and quantification of fibrosis, fat and fatty fibrosis in human left atrial myocardium using *ex vivo* MRI

**DOI:** 10.1371/journal.pone.0205104

**Published:** 2018-10-08

**Authors:** Khaoula Bouazizi, Amer Rahhal, Slawomir Kusmia, Morgane Evin, Carine Defrance, Philippe Cluzel, Myriam Berthet, Fabrice Atassi, Pascal Leprince, Guillaume Lebreton, Nadjia Kachenoura, Stéphane N. Hatem, Alban Redheuil

**Affiliations:** 1 Institute of Cardiometabolism And Nutrition, La Pitié-Salpêtrière Hospital, Paris, France; 2 Sorbonne University, INSERM 1146, CNRS 7371, Laboratoire d’Imagerie Biomédicale, Paris, France; 3 Sorbonne University, Faculté de médecine, UMRS 1166, Research unit on cardiovascular, metabolic and nutrition diseases, Paris, France; 4 Department of cardiovascular and thoracic surgery, Cardiology Institute, La Pitié-Salpêtrière Hospital AP-HP, Paris, France; 5 Department of cardiovascular imaging, interventional and thoracic radiology (DICVRIT), Cardiology Institute, La Pitié-Salpêtrière Hospital AP-HP, Paris, France; Universiteit Gent, BELGIUM

## Abstract

**Background:**

Atrial fibrillation is associated with an atrial cardiomyopathy composed mainly of fibrosis and adipose tissue accumulation. We hypothesized that MRI, when used in an optimal *ex vivo* setting allowing high spatial resolution without motion artifacts, can help characterizing the complex 3D left atrial (LA) wall composition in human myocardial samples, as compared to histology.

**Methods:**

This prospective case-control study was approved by the institutional review board. 3D MRI acquisitions including saturation-recovery T1 mapping and DIXON imaging was performed at 4.0 T on 9 human LA samples collected from patients who underwent cardiac surgery. Histological quantification of fibrosis and fat was obtained. MRI T1 maps were clustered based on a Gaussian Mixture Model allowing quantification of total, interstitial and fatty fibrosis components. Fat maps were computed from DIXON images and fat fractions were calculated. MRI measurements were performed on the same location as the histological analysis (plane) and on the entire sample volume (3D).

**Results:**

High correlations and levels of agreement were observed between MRI and histology for total (r = 0.93), interstitial (r = 0.93) and fatty fibrosis (r = 0.98) and fat (r = 0.96). Native T1 correlated with the amount of fibrosis from MRI and histology. The 3D MRI total, interstitial and fatty fibrosis ranges were between 6% and 23%, 4% and 17.3%; and 1.4% and 19.7% respectively.

**Conclusion:**

High Field *ex vivo* MRI was able to quantify different LA myocardial components with high agreement in 2D with histology and moreover to provide 3D quantification of such components whereas *in vivo* application remains a challenge.

## Introduction

Atrial fibrillation (AF) is the most common arrhythmia worldwide and a major cause of morbidity and mortality [1][2]. Most often, AF is associated with a profound alteration of the functional and structural properties of the left atria (LA) leading to a true cardiomyopathy [3][4]. Fibrosis and particularly fibro-fatty infiltration of the atrial myocardium are major determinants of this LA cardiomyopathy and its progression is responsible for increased myocardial stiffness and poor clinical outcome [3]. A relationship between impaired atrial strain and fibro-fatty atrial wall infiltration has been demonstrated *in vivo* by MRI [5]. Subsequently, accurately quantifying the amount of adipose tissue and fibrotic remodeling of the human atria is thus a crucial issue. Histology is the gold standard method to quantify myocardial adipose tissue and fibrotic components within the LA wall *ex vivo* at the microscopic level.

Recently, magnetic resonance imaging (MRI) T1 mapping, which enables quantification of the myocardial signal directly on a standard scale, has generated great interest as it allows *in vivo* quantitative myocardial tissue characterization non-invasively [6][7][8], potentially alleviating the need for biopsy. Indeed, at a macroscopic level, native T1 values, although non-specific, have been found to be sensitive markers of the presence and severity of disease [9][10][11]. However, to date this technique is largely restricted to the left ventricle (LV) because of partial volume issues related to relatively limited spatial resolution, thin LA myocardial wall and motion issues as well as near wall flow artifacts. The LA wall is very thin (2 mm) in comparison to the LV wall (8 to 11 mm) since its average thickness of 2 mm is only two to threefold the optimal spatial resolution achieved in *in vivo* MRI. Accordingly, it is very challenging to reliably measure T1 values within the LA wall without being subject to partial volume effects [12]. Few attempts with T1 mapping techniques have been made to quantify the degree of LA myocardial fibrosis noninvasively [13]. However, similar to late gadolinium enhancement (LGE) of the LA wall, whether such MRI measurements exclusively reflect fibrosis is being debated [14][15].

The aim of this work is to use MRI in an optimal *ex vivo* setting (high spatial resolution, no motion and flow artifacts, and no blood signal) to study the ability of MRI to characterize complex fibrotic and fat components of the LA wall. The DIXON method has been proposed to generate fat maps of the myocardium [16] and T1 mapping techniques have emerged recently to quantify myocardial fibrosis. In this study, we combined T1 mapping and the DIXON method to quantify fatty fibrosis in human LA myocardium. Furthermore, after 2D validation against histology, 3D volumetric computation of fibrosis and adipose tissue from MRI images was performed.

## Materials and methods

### Population

The institutional review board (Committee for the Protection of Persons, Paris EST) approved this prospective study and all participants gave signed informed consent. All adult patients undergoing open surgery for mitral regurgitation without cardio-circulatory assist devices were eligible. Exclusion criteria were contraindications to cardiac MR imaging and impossibility to perform cardiac MR imaging in the 24 hours prior to cardiac surgery. Eleven different tissue samples of the left atrium were collected from five adult patients undergoing cardiac surgery in our cardiac surgery department. Patients were included consecutively between January 2014 and March 2015. Seven samples were from one patient who underwent heart transplantation for refractory heart failure secondary to extensive anterior myocardial infarction and the remaining samples were collected from four patients who underwent mitral valve replacement for mitral valve regurgitation. Two samples were excluded due to inappropriate myocardial sample size which resulted in a very low signal to noise ratio in MRI images, and 9 samples were suitable for analysis.

### Tissue samples

During open cardiac surgery, a tissue sample was taken from the left atriotomy border performed on the left atrium inferior-medial wall between the pulmonary veins. Samples were fixed in neutral buffered paraformaldehyde 4% at room temperature and sent for MRI before histological analysis. MRI and histology quantitative analyses were blinded to each other.

### MR image acquisition

MRI was performed on a 4.0 T scanner (Bruker BioSpin MRI GmbH) equipped with a Bruker gradient insert with a coil of 2.8 cm in diameter.

Atrial tissue samples were immersed in fluorinert® FC-770 (Sigma-Aldrich, Steinheim, Germany) to avoid susceptibility artifacts and positioned in small tubes. Localized first and second-order shimming and frequency adjustment were performed to minimize off-resonance as off-resonance causes both regional and global underestimation of T1.

### T1 and T2 imaging

A Rapid Acquisition with Relaxation Enhancement (RARE) sequence was implemented with different TR delays. This sequence is relatively insensitive to susceptibility mismatch, it thus produces good images at high field [17] without requiring extensive shimming time. Imaging parameters were as follows: FOV (Field-of-view) = 3.84 cm x 1.92 cm, slice thickness: 1 mm, BW (Bandwidth)/pixel = 840 Hz, 3 slices, TE (echo time) = 11.4 ms, repetition time (TR) array (s): 0.15; 0.25; 0.4; 0.65; 1.0; 1.5; 2.5; 4.0; 6.5, reconstruction matrix = 192 x 96 and number of excitations (NEX) = 2, linear profile ordering, in-plane spatial resolution was 200 x 200 μm. A radiofrequency fat saturation pulse was used to delete any signal from spins related to adipose tissue.

T2 maps were generated from MSME (multi-slice multi-echo) images after 48 echoes assuming a monoexponential transverse decay. Acquisition parameters were: TE/TR = 0.01 ms / 6.5 s, 5 slices, FOV = 3.84 cm x 1.92 cm, slice thickness: 1 mm, matrix: 192 x 96, in-plane spatial resolution was 200 x 200 μm.

### Fat imaging

A conventional Cartesian gradient echo (Fast Low Angle Shot: FLASH) sequence with multi-echo readout routinely used in MRI was performed for detection and quantification of fat with the following parameters: FOV = 3.84 cm x 1.96 cm, TE (ms) = 3.356; 4.194; 5.033, TR = 0.19 s, flip angle: 30°, 5 slices, slice thickness: 1 mm, NEX = 8, BW/pixel = 840 Hz, matrix: 192 x 96, in-plane spatial resolution was 200 x 200 μm.

### MR image analysis

#### T1 mapping and fibrosis quantification

Reconstruction of T1 maps from RARE images was performed off-line using a custom software written in Matlab (The MathWorks, Natick, MA, USA). After reordering source images according to their TR and merging them into one dataset, T1 values were computed for every pixel by three-parameter nonlinear curve fitting resulting in a parametric T1 map:

$$S=A\;\left(1-B\;\exp\left(\frac{-t}{T1}\right)\right)$$ (1)

where ***S*** corresponds to signal amplitude, ***t*** to repetition delay, ***A*** and ***B*** to additional fit parameters. The three-parameter model for data fitting was proposed to make T1 measurements less sensitive to T2 effect and saturation efficacy and to reduce T1 estimation error [12].

Fibrosis was characterized according to T1 values from T1 maps. A classification method based on a Gaussian Mixture Model (GMM) [18] was implemented in Matlab (The MathWorks, Natick, MA, USA) and was used to cluster tissue components. The GMM assumes a Gaussian distribution of the T1 value of each fitted class, each class having its own mean intensity and variance. To separate myocardial components, the number of classes for the GMM algorithm was set to three (myocardium, fibrosis and PFA (Paraformaldehyde)) to compute total fibrosis and to four (myocardium, a mixture of fat and fatty fibrosis, interstitial fibrosis and PFA) to compute interstitial fibrosis. Fibrosis was selected as the GMM class with higher mean T1 value than myocardial class and lower than PFA class. Fat maps and T1 maps were superimposed and the non-fat region was eliminated in order to generate a T1-fat map. To compute fatty fibrosis the T1-fat map was subsequently clustered into two classes (fat and fatty fibrosis). The fibrosis fraction was computed for each slice and for the volume (averaged over three slices).

### Fat imaging

By exploiting the chemical shift between fat and water protons (≈600 Hz at 4.0 T), one can acquire in-phase (IP) and out-of-phase (OP) magnitude images by varying the echo time. A dual-echo FLASH sequence was run and in-phase image was acquired at TE = 3.356 ms where the signals from water and fat protons are summed constructively and at TE = 4.194 ms where the signal from water and fat protons destructively interferes. The IP image is the sum of water and fat signal and the OP image is the difference between water and fat signal. Accordingly the fat fraction is defined as:

$$FF=\frac{IP-OP}{2\;IP}$$ (2)

To correct for T2* decay, fat maps were computed as proposed by Liu *et al*. [16] while assuming the same T2* for fat and water. Fat fractions were computed for every pixel by using Eq (2) resulting in a parametric fat map.

A pixel-wise myocardial T2* map was generated for each sample using a monoexponential fit on the magnitude MSME data where a Rician noise distribution was assumed. MRI fat fractions were calculated for each slice and then summed and averaged over the acquired slices (five slices) resulting in fat volume fraction for each sample.

### Histological analysis

After MRI, LA samples were embedded in paraffin for histological study. They were cut in 6 μm-thick sections and stained with 0.1% Picro-Sirius Red for histological evaluation of fibrosis and adipose tissue on a single slice in the same slice orientation as used for MRI.

Slices were digitized in nd2 format using a high quality resolution technique with an ECLIPSE TiE inverted microscope (Nikon Corporation, Tokyo, Japan). All scanned slices were taken with magnification of an X10 objective lens.

Histological image processing was performed using HistoLab version 10.2.1 (Microvision Instruments, Evry, France). Quantification of fibrosis and adipose tissue from histological images was performed using semi-automated segmentation through multi-channel thresholding method based on the color and illumination contents. Adipose tissue, fibrosis and myocardium were assessed visually. The histology images were first manually determined by outlines, which defined each tissue component separately. We attributed red color for fibrosis and yellow for myocardium then we determined the saturation and intensity of colors on the overall sections allowing quantification of the different tissue components. Artifacts and objects of non-interest (background, perivascular, endocardial fibrosis, and epicardial fibrosis) were manually delineated from the selected area [19].

The quantitative results were expressed as the surface area in mm^2^ for myocardium, fibrosis and adipose tissue. We defined in percentage the extent of total, interstitial and fatty fibrosis in each slice as the ratio of the respective total and interstitial and fatty fibrosis surface area divided by the total tissue surface area x 100. We defined the extent of adipose tissue in the slice as the ratio of the adipose tissue surface area to the total tissue surface area: adipose tissue fraction (%) = (area of adipose tissue/total tissue area) × 100.

### Statistical analysis

Statistical analysis was performed using GraphPad PRISM 6 (GraphPad Software Inc., Canada). Data are presented as percentage values according to the total surface of slices for both interstitial, fatty and total fibrosis and adipose tissue in histological and MRI studies. Linear regression was used to compare the percentage of fibrosis and adipose tissue between histology and MRI. The Spearman’s rank correlation coefficient was used to report the relationship between MRI and histological findings. Significance of the difference between T1 and the different left atrial components correlations was evaluated using an Hotelling–Steiger test [20]. To test the effect of lowering spatial resolution to comply with *in vivo* acquisitions, spatial resolution of our maps was changed between the acquired value of 200 μm and 2 mm and the percentage of variation of the amount of the different tissue components was calculated. Percentage of variation was defined as the difference between the two measurements divided by the reference measurement (at 200 μm). The significance was set to *P* < 0.05 for all tests. Bland-Altman analysis was used to assess agreement between MRI and histology and mean difference as well as corresponding 95% confidence intervals (±1.96 SD) were reported.

## Results

### Visual comparison between atrial wall components in MRI and histology

**Fig 1:**
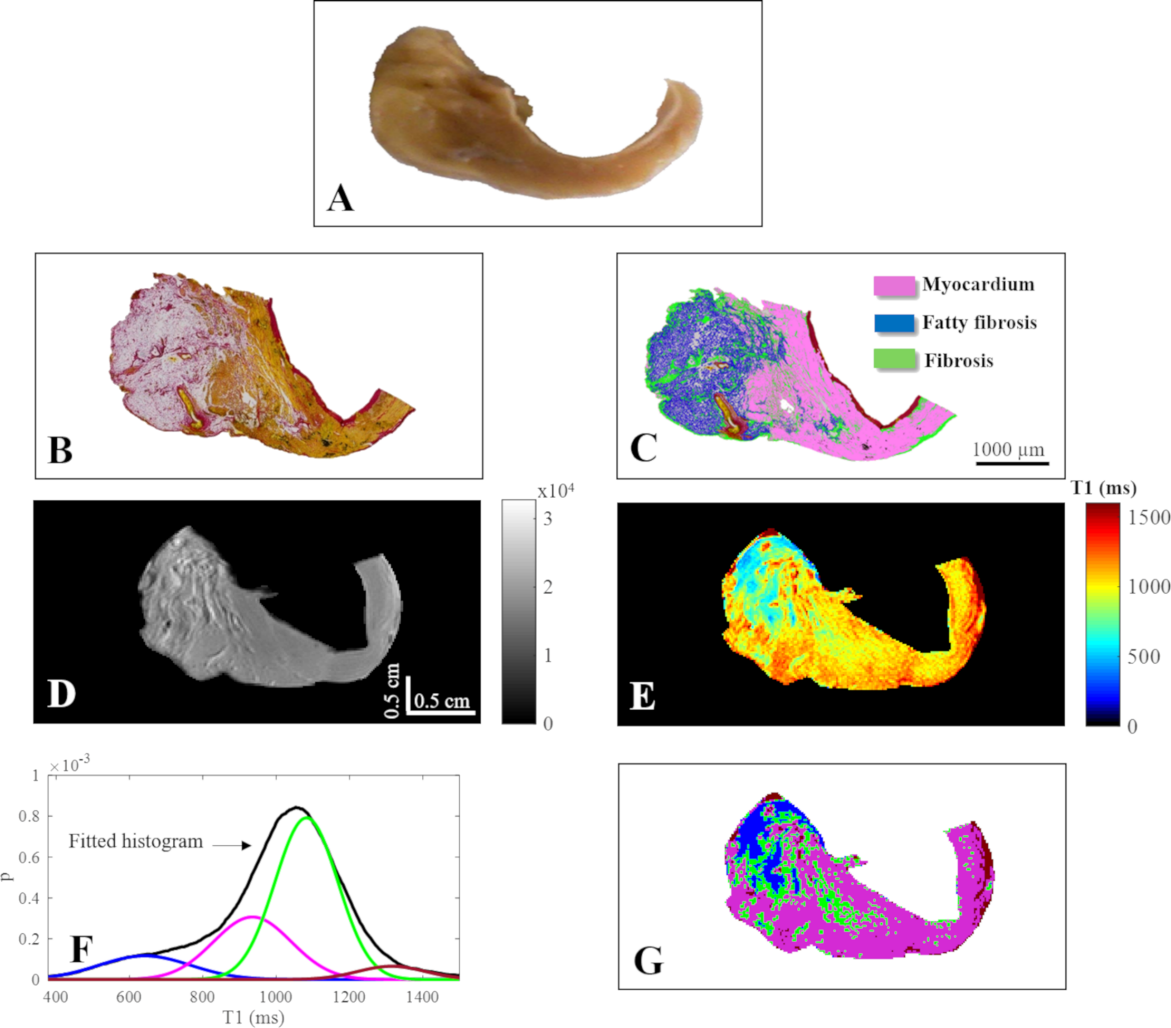
Representative left atrial sample (histology and MRI). (A) Representative left atrial sample (macroscopy), (B) photomicrograph of the Picro-Sirius Red stained section showing adipose tissue, fibrotic remodeling and myocardium and (C) semi-automated quantitative analysis identifying fibrosis (green), fibrotic changes in adipose tissue (blue), and myocytes (pink); 10X magnification, scale bar 1000 μm. (D) RARE image (colorbar in arbitrary unit) of the same sample acquired at TE/TR = 11.4 ms/6.5 s, FOV = 3.84 cmx1.92 cm, matrix = 192x96, (E) T1 map, (F) T1 histogram with 4 Gaussian distributions and the corresponding clustering map (G) where fat appears in blue, myocardium in pink, interstitial fibrosis in green and PFA in dark red.

In order to study whether MRI was able to distinguish the various histological components of the atrial wall, we compared images of the same atrial section after Picro-Sirius Red staining and MRI imaging in nine LA myocardial samples. We found a very good correspondence between MRI and histology images. Fig 1 illustrates a macroscopic LA sample (Fig 1A), the corresponding histological image (Fig 1B) and its quantitative analysis (Fig 1C) and reveals the high spatial matching of the different atrial wall components with MRI. Fig 1D and 1E shows a RARE image of the same LA sample and corresponding T1 map. Fig 1F and 1G show T1 values fitted histogram with four Gaussian distributions and the corresponding GMM clustering map.

### GMM clustering

In all examined samples, the fibrosis class peak was the highest and has the narrowest FWHM (full width at half maximum) compared to other classes (Table 1). The variability of the FWHM of the fibrosis class was also the lowest. After increasing the number of classes from 3 to 4 accounting for the fatty component, more precision on the fibrosis class was observed. Indeed, the FWHM of the fibrosis class became narrower (from 288±42 ms to 219±16 ms) and its variation decreased (from 14% to 6%). The number of reclassified pixels for the fibrosis class differed between samples and between slices. The percentage of such variation was in the range 0.1% - 28% of the total number of pixels in each sample.

**Table 1:** Peaks and FWHM averaged over all the human left atrial samples (mean±SD).

	**3 classes**			
	**myocardium**	**total fibrosis**	**PFA**	
**peak (au)**	1.19±1	5.22±2.02	0.7±0.55	
**FWHM (ms)**	480±268	288±42	490±67	
	**4 classes**			
	**myocardium**	**fat+fatty** **fibrosis**	**Interstitial fibrosis**	**PFA**
**peak (au)**	0.56±0.75	2.15±1.39	5.55±1.7	1.23±0.96
**FWHM (ms)**	424±225	222±64	219±16	590±43

### Quantitative comparison of atrial fibrosis in MRI and histology

**Fig 2:**
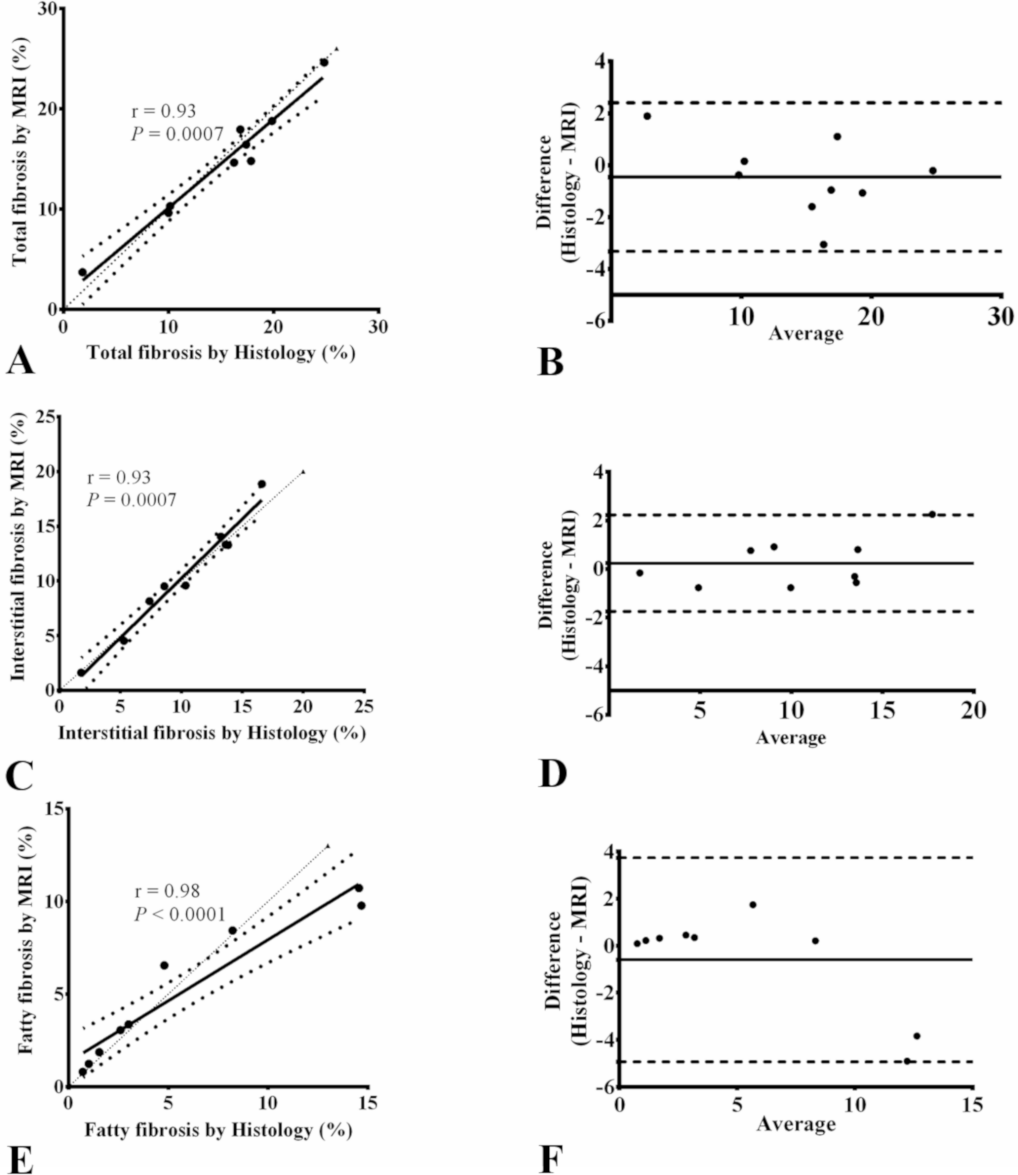
Spearman correlations of total, interstitial and fatty fibrosis between MRI and histology and the corresponding Bland-Altman plots. Linear regression plots for comparisons between MRI and histology in terms of total (A), interstitial fibrosis (C) and fatty fibrosis (E) fractions and the corresponding Bland-Altman plots: total (B), interstitial fibrosis (D) and fatty fibrosis (F) fractions. In Bland-Altman plots, the thick solid line represents the bias and dashed lines represent 95% confidence intervals. The thin dotted line in regression plots represents the identity line.

Next, we studied the ability of MRI to quantify atrial fibrosis subtypes (total, interstitial and fatty). Fig 2 shows a high correlation (r = 0.93, *P* = 0.0007) between MRI and histology in terms of total fibrosis fraction. The Bland-Altman analysis showed good agreement between the two measurements as revealed by a mean difference of -0.46% and 95% limits of agreement from -3.32% to 2.4%. Interstitial fibrosis fraction measured from MRI was also highly correlated to histology (r = 0.93, *P* = 0.0007) and Bland-Altman analysis revealed excellent agreement as shown by a mean difference of 0.24% and 95% limits of agreement from -1.75% to 2.23%. Fatty fibrosis fraction from MRI was also highly correlated to histology (r = 0.98, *P*<0.0001) and Bland-Altman analysis revealed very good agreement as shown by a mean difference of -0.59% and 95% limits of agreement from -4.94% to 3.74%.

The fibrosis fraction computed in the whole 3D volume from MRI ranged between 6% and 23% for total fibrosis, between 4% and 17.3% for interstitial fibrosis, between 1.4% and 19.7% for fatty fibrosis fractions. Correlations between 3D and 2D measurements were r = 0.76 (*P* = 0.02) for total fibrosis fraction, r = 0.81 (*P* = 0.01) for interstitial fibrosis fraction and r = 0.60 (*P* = 0.09) for fatty fibrosis fraction.

**Fig 3:**
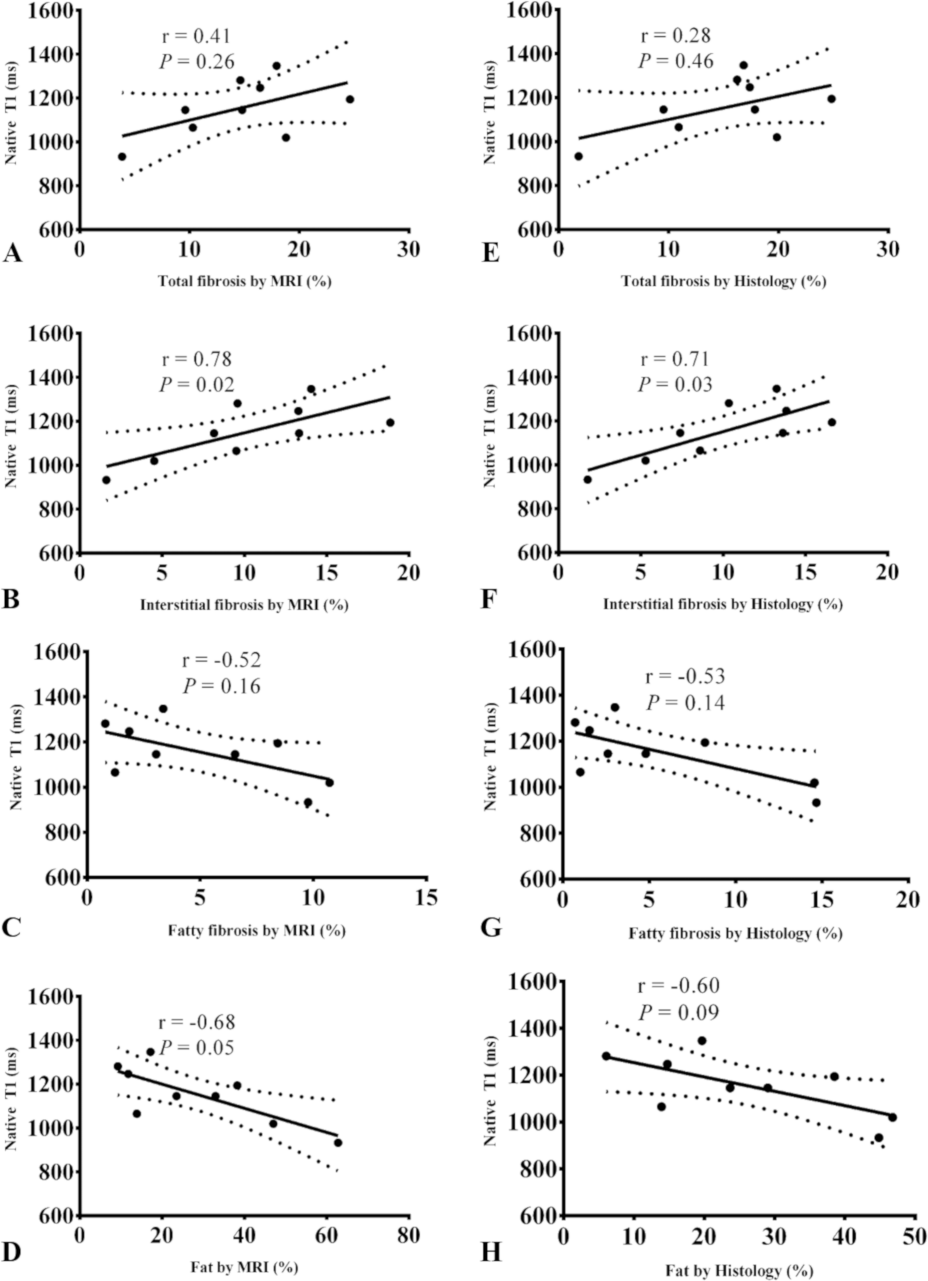
Spearman correlations between native T1 and different left atrium components from MRI and histology. Linear regression between mean myocardial native T1 values and 2D total, interstitial and fatty fibrosis and fat fractions measured from MRI (A-D) and histology (E H).

Mean native T1 values measured in all samples ranged between 923 and 1285 ms (1154±124 ms). In all samples, fibrotic areas presented higher native T1 values (1131±89 ms) than non-fibrotic myocardium (T1 = 962 ± 21 ms). Native mean T1 values presented a significant and positive correlation with interstitial MRI fibrosis fraction (r = 0.41, *P* = 0.01) and trends for a positive correlation but not significant with total (r = 0.41, *P* = 0.26) MRI fibrosis fraction (r = 0.78, *P* = 0.01) and a negative correlation with fatty fibrosis (r = -0.52, *P* = 0.16) and fat MRI fraction (r = -0.68, *P* = 0.05) (Fig 3A and 3D). Native mean T1 values presented a significant and positive correlation with interstitial histological fibrosis fraction (r = 0.71, *P* = 0.03) but not with total histological fibrosis (r = 0.28, *P* = 0.46) and a negative trend of correlation with fatty fibrosis (r = -0.53, *P* = 0.14) and fat histological fraction (r = -0.60, *P* = 0.09) (Fig 3E and 3H). Overall, the higher correlation coefficient was found between native T1 and interstitial fibrosis fraction from MRI (Fig 3B), however this did not reach statistical significance (*P* = 0.07).

### Quantitative comparison of atrial adipose tissue in MRI and histology

**Fig 4:**
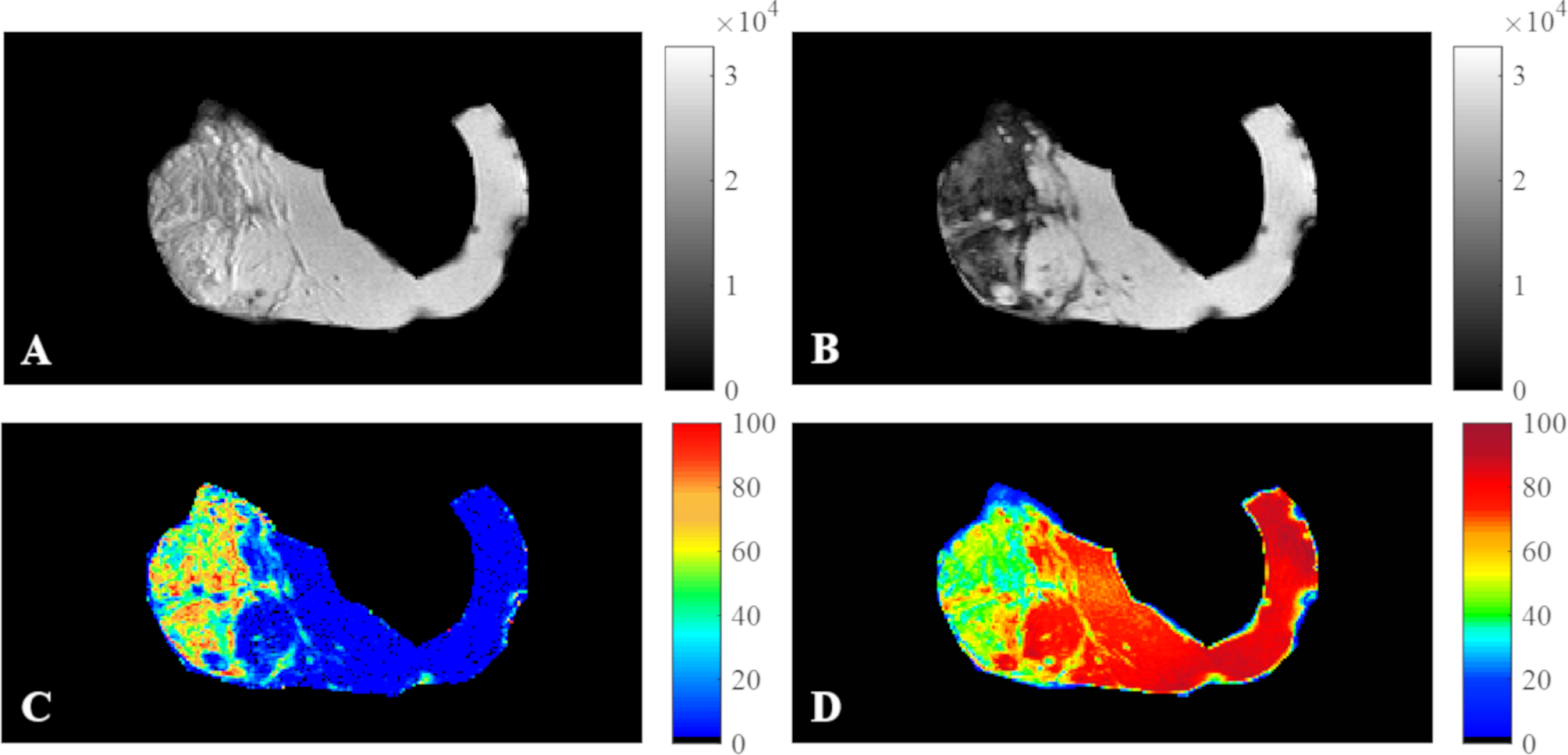
In-phase, out-of-phase images, fat and water maps of a left atrial sample. In-phase (A), out-of-phase images (B), fat (C) and water (D) normalized maps of the LA sample presented in Fig 1. Fat fraction from histology = 46.8% and from MRI = 47%.

**Fig 5:**
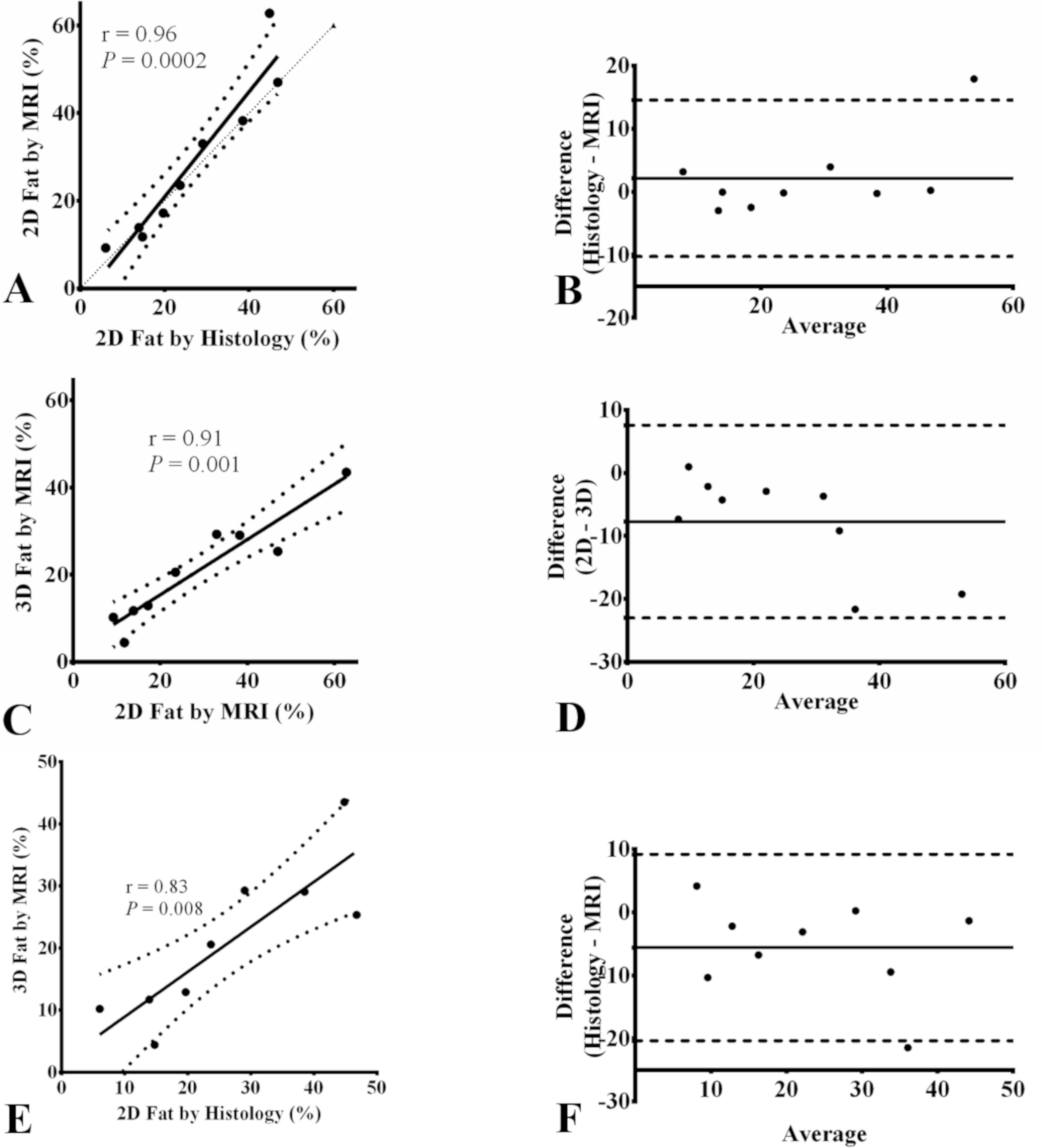
Spearman correlations of total, interstitial and fatty fibrosis between MRI and histology and the corresponding Bland-Altman plots. Linear regressions for comparison between MRI and histology in terms of 2D fat fraction (A), between 3D fat by MRI and 2D fat by histology (C) and between 3D fat by MRI and 2D fat by histology €; and the corresponding Bland-Altman plots (B,D,F). The thin dotted line in regression plots represents the identity line. In Bland-Altman plot, the thick solid line represents the bias and dashed lines represent 95% confidence intervals.

Fat fractions computed from MRI fat maps (Fig 4) were strongly correlated (Fig 5A) with histological fat fractions (r = 0.96, *P* = 0.0002). Bland-Altman analysis (Fig 5B) revealed a good agreement between the two measurements as shown by a mean difference of 2.14% and 95% limits of agreement from -10.27% to 14.55%. 3D fat fraction values from MRI ranged between 4.4% and 43% and correlated well with 2D fat fractions (r = 0.92, *P* = 0.0013) as well as histological fat (r = 0.83, *P* = 0.008) (Fig 5C and 5F). The distribution of different left atrium components in each examined sample is detailed in S1 Fig.

### Spatial resolution and tissue characterization *in vivo*

Fibrosis and fat amounts from MRI images with decreased spatial resolution (2 mm) were computed. When decreasing the spatial resolution to levels achieved *in vivo*, fibrosis amount was underestimated and fat amount overestimated. The percentage of variation of total fibrosis between the acquired resolution (200 μm) and the simulated resolution (2 mm) was between -3% and -53% (-16±15%). The percentage of variation of interstitial fibrosis was between -6% and -26% (-14±8%). The percentage of variation of fatty fibrosis was between -1% and -50% (-28±17%). The percentage of variation of fat was between +6% and +63% (+30±21%).

## Discussion

Our results revealed that when MRI T1 mapping and fat imaging are used in the optimal *ex vivo* settings, with no motion or flow artifacts, high spatial resolution and free of blood signal, they are able to accurately characterize different myocardial components at the tissue level including total fibrosis, interstitial fibrosis, adipose tissue and fibrotic fat in the human let atrial wall. Indeed, highly significant correlations were found for the aforementioned tissue components when comparing MRI quantitative measurements to histology. Furthermore, the consistency between MRI 2D and 3D quantitative measurements indicates the ability of MRI to quantify the myocardial tissue components in the entire sample volume, which is hardly obtainable from histology. Accordingly, to the best of our knowledge, this is the first study to analyze human left atrial myocardium *ex vivo* and to demonstrate the ability of MRI to successfully identify the complexity of fibro-fatty infiltration in LA myocardium. Such quantitative characterization would be very relevant *in vivo* to LA cardiomyopathies since atrial structural remodeling is associated with irreversible alterations in atrial mechanical and electrical properties [4], ultimately leading to AF and adverse outcome.

The amount of fibrosis in LA samples was quantified from T1 maps computed from a RARE sequence at 4.0 T. Although RARE is relatively prone to artifacts since images generally have low signal to noise ratio, it was chosen because of high accuracy for T1 measurement and less sensitivity to magnetization transfer [21] as well as to T2 effect. A three-parameter model was used to fit the recovery signal. This model was shown to capture the effects of the recovery curve disturbance caused by multiple RF excitations before the *k*-space center as well as to reduce the susceptibility to magnetization transfer [21][22]. The GMM technique was chosen for clustering as a simple and unsupervised approach. Indeed, only the number of clusters is needed to initialize the GMM technique.

In all LA samples, fibrotic areas presented higher T1 than non-fibrotic myocardium and in the same range as cited in other *in vivo* observations at close field strength [23][24][25], although direct comparison of T1 values is not possible because of differences in acquisition pulse sequences and field strength between different studies. The increase in myocardial T1 values corresponds to the course of the fibrotic infiltration mixed with inflammatory processes. Elevated myocardial T1 of the left ventricular wall has been reported in a number of commonly encountered cardiac conditions including myocarditis, hypertrophic and dilated cardiomyopathy, myocardial infarction, amyloidosis, and diffuse fibrosis in patients with aortic valve stenosis [6][9][10][26][27].

Consistency of our T1 measurements was also demonstrated by the significant positive correlation with histological interstitial fibrosis fraction, which was higher or in the same range with correlation values reported by *in vivo* studies analyzing left ventricular myocardial biopsies in subjects undergoing aortic valve replacement (r = 0.65) [27] and in DCM patients (r = 0.67) [28]. Of note, in *in vivo* studies, the presence of post-contrast T1 maps allows for the calculation of the extracellular volume which has also been correlated to histological fibrosis fraction [25][29][30]. The strongest correlate of native myocardial T1 in this study was found to be the degree of interstitial fibrosis, either from MRI or histology, which confirms the potential of T1 as a sensitive indicator of extracellular myocardial alteration in the absence of any blood signal and artifacts. Indeed, free water molecules related to fibrosis (extracellular space expansion) present a fast tumbling (molecular motion). Expansion of the collagen matrix increases the volume of distribution leading to native T1 augmentation.

We found native T1 to be inversely related to the extent of adipose tissue as the T1 of fat is lower than that of myocardium or fibrosis (slow tumbling of fat large molecules). Consequently, fatty fibrosis was also negatively related to T1 as the signal from the fat component (low T1) prevailed over the signal from fibrosis (high T1). This can explain the absence of significant positive relationship between total fibrosis and native T1 over the whole sample *ex vivo*. This issue has been raised by Kellman *et al*. [31] and underlines the potential confounding effect of fat on the relationship between fibrosis and T1 *in vivo*. Adipose tissue could impact T1 values (when there is a mixture of water and fat in the myocardium) for the quantification of fibrosis, since adipose tissue will lower the native T1 compared to fibrosis which increases native T1 value. An ideal setting would be a complete set of T1, T2, B0 and water/fat separated images acquisitions for comprehensive tissue characterization, but the acquisition time for all these datasets remains prohibitive *in vivo*.

The small samples size may also explain the variations in the correlations observed in our study compared to other studies. Bull *et al*. [27] observed a good correlation between native T1 values and collagen volume fraction in 19 patients from histology (r = 0.65, p = 0.002). Goto *et al*. [28] reported a better correlation in 20 patients (r = 0.673, p = 0.001). Caudron *et al*. [23] observed the best correlation (r = 0.71, p<0.001) in 75 mice. According to these results, one can expect a stronger correlation when including more samples. Also, samples from the literature concerned left ventricular myocardium in which the fat component is more modest and a potentially less confounding tissue component and rodent models are also known to have very little myocardial fat. The purpose of our study was to demonstrate the potential confounding effect of fat on the global atrial myocardial T1 even in high resolution *ex vivo* imaging making *in vivo* quantification of fibrosis based on T1 hazardous.

2D fat fractions computed from the FLASH sequence were highly correlated to histological fat fractions. To measure proton density of fat fraction accurately, we accounted for B0 field inhomogeneity (the samples were immersed in fluorinert), and T2* decay [16]. Magnitude images were used to quantify fat fraction because they have been shown to produce unbiased estimates in the presence of phase errors due to eddy currents in the acquired echoes [32]. Here we also quantitatively report and validate *versus* histology not only the degree of fat in the overall myocardium but the extent of fibro-fatty infiltration which has been specifically incriminated in playing a pivotal role in the development of LA cardiomyopathy irrespective of the underlying disease [3][33].

*In vivo* assessment of myocardial fat has been proposed by spectroscopy or imaging [16] but the quantitative study of the fibrotic component in overall left atrial myocardium or moreover fibrosis within the myocardial fat requiring reliable T1 mapping remains a major challenge *in vivo* when applied to the thin (2 mm) left atrial wall [13]. Improvements in spatial resolution may in the future allow the translation of the *ex vivo* results to patients in the clinical setting and uniquely provide compartmental quantitative tissue characterization of the LA wall *in vivo*. Such data at the tissue level would fill an important gap in currently available information *in vivo* as LA biopsies are not performed and could increase our understanding of LA cardiomyopathy and pave the way for novel management strategies.

This study has several limitations. First, there is a relatively small sample size. This reflects the high difficulty to obtain tissue samples in patients. Second, it is not currently possible to apply the *ex vivo* T1 mapping sequence used here *in vivo* in a moving heart with a sufficient spatial resolution to reliably study the thin left atrial wall. However, our primary goal was here to demonstrate the ability of MRI to characterize the complex LA myocardial compartments and define the determinants of native T1 in “ideal” conditions. This technique may be immediately useful in analyzing myocardial biopsies to reduce sampling errors or in animal validation studies in the whole heart before technical improvements make them ready for translation to *in vivo* human studies. Further studies should be performed to overcome the challenging technical issues which will allow translation to a moving heart.

## Conclusion

T1 mapping combined with DIXON MRI sequences acquired *ex vivo* on human LA myocardium provided highly accurate quantification of myocardial components including fibrosis, fat and fatty fibrosis, as revealed by highly significant correlations with histology. Increased native T1 was strongly related to increased interstitial fibrosis and the extent of adipose tissue tended to decrease T1 values. Furthermore, MRI was able to propose a 3D distribution of these myocardial tissue components over the sample.

## Supporting information

S1 Fig Left atrium composition of different examined left atrial samples. (DOCX)
