# Supplementary material for: Differentiation and quantification of fibrosis, fat and fatty fibrosis in human left atrial myocardium using ex vivo MRI
Source: PLoS One. 2018 Oct 8;13(10):e0205104. doi: 10.1371/journal.pone.0205104 (PMC6175289; doi:10.1371/journal.pone.0205104)
Supplement: S1 Fig — (DOCX) [file pone.0205104.s001.docx]

# **Supporting Information**

The combination of 3D fibrosis and fibro-fatty percentages in all samples ranged between 8% and 27% which are close to the 3D total fibrosis percentage (6% - 23%). The small difference between intervals originates from two samples containing a very high amount of fat (>40%) with fibrotic infiltration. A fibrosis difference between slices may reach 20% depending on the orientation of the slice and depending on each slice. Moreover, 2D imaging may suffer from sampling bias depending on the sample orientation, size and fibrosis and fat composition.

Figure I shows the distribution of different left atrium components in each examined sample. It seems that interstitial fibrosis amount of 10±5% and/or fatty fibrosis amount of 5±3.7% and/or fat amount of 28±18% may cause a structural and function impairment of left atrium.

Figure I. Left atrium composition of different examined left atrial samples.
